# LPS Guides Distinct Patterns of Training and Tolerance in Mast Cells

**DOI:** 10.3389/fimmu.2022.835348

**Published:** 2022-02-17

**Authors:** Marco De Zuani, Chiara Dal Secco, Silvia Tonon, Alessandra Arzese, Carlo E. M. Pucillo, Barbara Frossi

**Affiliations:** ^1^ Department of Medicine, University of Udine, Udine, Italy; ^2^ International Clinical Research Center, St. Anne’s University Hospital Brno, Brno, Czechia

**Keywords:** mast cell, trained immunity, endotoxin tolerance, cytokines, LPS, *Candida albicans*

## Abstract

Mast cells (MCs) are tissue-resident, long lived innate immune cells with important effector and immunomodulatory functions. They are equipped with an eclectic variety of receptors that enable them to sense multiple stimuli and to generate specific responses according on the type, strength and duration of the stimulation. Several studies demonstrated that myeloid cells can retain immunological memory of their encounters – a process termed ‘trained immunity’ or ‘innate immune memory’. As MCs are among the one of first cells to come into contact with the external environment, it is possible that such mechanisms of innate immune memory might help shaping their phenotype and effector functions; however, studies on this aspect of MC biology are still scarce. In this manuscript, we investigated the ability of MCs primed with different stimuli to respond to a second stimulation with the same or different ligands, and determined the molecular and epigenetic drivers of these responses. Our results showed that, while the stimulation with IgE and β-glucan failed to induce either tolerant or trained phenotypes, LPS conditioning was able to induce a profound and long-lasting remodeling of the signaling pathways involved in the response against LPS or fungal pathogens. On one side, LPS induced a strong state of unresponsiveness to secondary LPS stimulation due to the impairment of the PI3K-AKT signaling pathway, which resulted in the reduced activation of NF-κB and the decreased release of TNF-α and IL-6, compared to naïve MCs. On the other side, LPS primed MCs showed an increased release of TNF-α upon fungal infection with live *Candida albicans*, thus suggesting a dual role of LPS in inducing both tolerance and training phenotypes depending on the secondary challenge. Interestingly, the inhibition of HDAC during LPS stimulation partially restored the response of LPS-primed MCs to a secondary challenge with LPS, but failed to revert the increased cytokine production of these cells in response to *C. albicans*. These data indicate that MCs, as other innate immune cells, can develop innate immune memory, and that different stimulatory environments can shape and direct MC specific responses towards the dampening or the propagation of the local inflammatory response.

## Introduction

Despite lacking the fine antigen specificity, clonality, and longevity of adaptive lymphocytes, innate immune cells have been demonstrated to retain traits of memory of earlier challenges (e.g., infection or vaccination) and thereby display increased responsiveness upon re-challenge with the same or even unrelated stimuli (1). This enhanced state of immune activation is known as “innate immune memory” or “trained immunity” (2). Acquisition of a trained immunity state by innate immune cells involves metabolic, epigenetic and transcriptional reprogramming which is sustained for months (3, 4). Most studies have focused on changes in immune cell responses upon primary activation *via* numerous TLR ligands such as LPS, β-glucan, Pam3CSK4, CpG, and muramyl dipeptide, with training or tolerant effects depending on the type of the first stimulus received by the cells (5). This process has been described on many innate immune cells (such as monocyte/macrophages and natural killer cells) in both humans and rodents, but not completely for mast cells (MCs).

MCs belong to the innate branch of the immune system and are particularly abundant at sites where they are likely to encounter foreign molecules or microbial ligands. Both their terminal differentiation and responsiveness are highly influenced by tissue-specific stimuli. Moreover, MCs are fully equipped with an eclectic variety of receptors, and thus can be directly activated by a plethora of different stimuli (6). This plasticity, together with the strategic location in which they are normally found, makes it easy to understand why MCs are involved in different processes, from the defense against pathogens to the maintenance of tolerance and the crosstalk with other cells of the adaptive immune system (7). This versatility depends on the ability of MCs to detect pathogens and danger signals and release a unique panel of mediators to promote pro- and anti-inflammatory reactions (8).

Given the ubiquitous tissue presence of MCs and their capability to respond to a broad spectrum of stimuli, it is expected that MCs, as other innate immune cells, may also retain innate immune memory, which could explain the diverse contribution of these cells in different physiological and pathological settings (9). Consistent with this hypothesis, it has been recently demonstrated that MCs can retain a memory phenotype as a consequence of epigenetic modifications that regulate their differentiation and response to danger signals (10). Similarly, stimulation with LPS is known to induce a transient unresponsiveness in MCs (11), while IgE sensitization was described to increase their responsiveness to LPS (12). Notably, both positive and negative effects of LPS priming on FcεRI-dependent activation of MCs have been documented, suggesting a critical role of LPS in MC-dependent allergic reactions (13). However, the development of a tolerant or trained phenotype in MCs has been poorly investigated and the studies conducted so far are based on short-term experiments, with the cells being restimulated and analyzed immediately after removal of the first stimulus.

In this manuscript, we aimed to determine the long-term effect of different immunological and microenvironmental stimuli on MCs development and reactivity, by adopting an experimental panel that allowed the cells to rest for 6 days between the first stimulation and the subsequent ones. The ability of MCs primed with different stimuli (LPS, curdlan, and IgE/Ag) to respond to a secondary stimulation with the same or different triggers, was investigated at molecular and epigenetic level revealing a specific behavior of the MC that depends on the type of signal the cell received.

## Material and Methods

### Mice

C57BL/6 mice were purchased from Envigo (Netherlands) and maintained at the animal facility of the Department of Medicine, University of Udine (Italy). All animal experiments were performed according to the animal care and in accordance to institutional guidelines and national law (D.Lgs. 26/2014).

### BMMCs Generation

Bone marrow-derived MCs (BMMCs) were obtained from 6- to 8- weeks-old mice by *in vitro* differentiation of bone marrow-derived progenitors obtained from mice femurs and tibiae. Precursor cells were cultivated in complete IL-3 medium [RPMI 1640 medium (Euroclone) supplemented with 20% FBS (Sigma Aldrich), 100 U/ml penicillin, 100 mg/ml Streptomycin, 2 mM Glutamine, 20 mM Hepes, non-essential aminoacids, 1 mM Sodium Pyruvate (Euroclone), 50 mM β-mercaptoethanol (Sigma Aldrich) and 20 ng/ml IL-3 (Peprotech)] at 37°C in 5% CO_2_ atmosphere. After 5 weeks, BMMCs differentiation was confirmed by flow cytometry by staining with anti-FcεRIα–PE (MAR-1) and anti-cKit–PE-Cy7 (ACK2) conjugated antibodies (eBiosciences). Data were acquired with a FACScalibur cytofluorimeter (Becton Dikinson) and analyzed with FlowJo software (FlowJo LLC). BMMCs were usually more than 98% cKit and FcεRI double positive.

### *Candida albicans* Cultures

Wild-type *Candida albicans* (*C. albicans*) SC5314 strain was a kind gift of Prof. Luigina Romani, University of Perugia (Perugia, Italy). Yeast was seeded on Sabouraud agar supplemented with 50 μg/ml chloramphenicol and incubated at 30°C for 24 hours. To generate *C. albicans hyphae*, 10^7^ yeast cells were resuspended in complete RPMI medium, seeded into T-25 adhesion flasks and allowed to germinate for 3 hours at 37°C. *Hyphae* were harvested by scraping, centrifuged at 700 x g for 10 minutes and washed with PBS.

### BMMCs Activation

Before all *in vitro* experiments, BMMCs were starved for 1 hour in IL-3–free complete RPMI medium. For IgE-dependent activation, BMMC were sensitized in IL-3–free complete RPMI medium for 3 hours with 1 μg/ml of dinitrophenol (DNP)- specific IgE, washed twice and challenged with 100 ng/ml DNP (Sigma- Aldrich).

For *C. albicans* infections, BMMCs were stimulated with *C. albicans* yeast (1:1 ratio) or *hyphae* (1:10 ratio) at a final concentration of 2 x 10^6^ cells/ml in IL-3–free complete RPMI medium. To limit fungal growth, Amphotericin-B (Sigma Aldrich) was added to each well at a final concentration of 10 ng/ml. RNA extraction was performed before the addition of Amphotericin-B.

**Figure 1:**
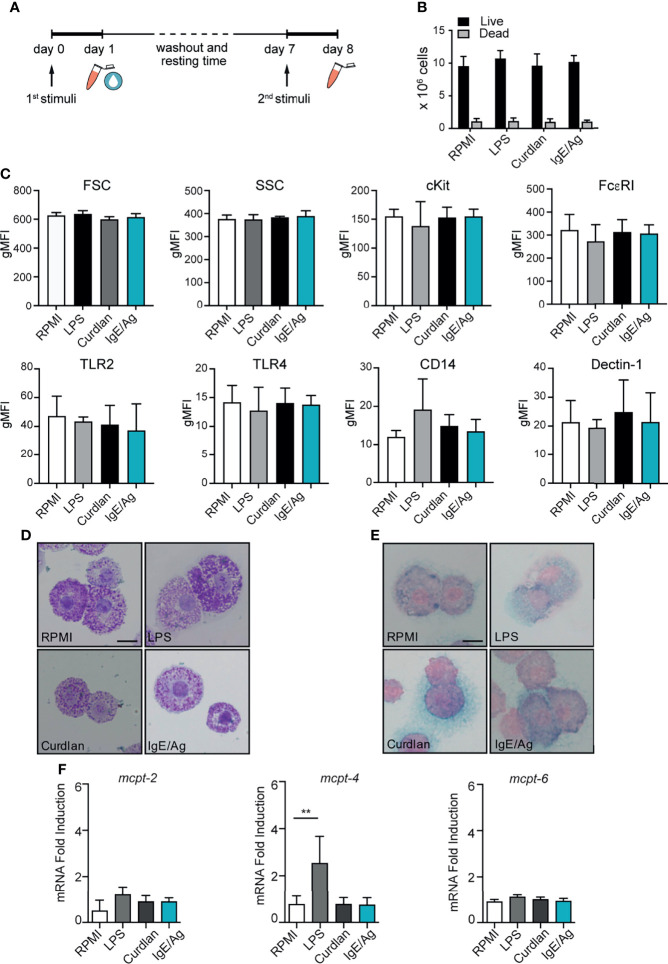
MC vitality and phenotype after the conditioning period. **(A)** Schematic representation of experimental protocol. BMMC were first stimulated for 24h with LPS, curdlan, IgE/Ag or left untreated (RPMI). Then cells were left to recover for 6 days, countedand tested for viability by means of trypan blue exclusion **(B)**, analyzed for surface marker expression **(C)**, stained with Toluidin blue **(D)** and Alcian-blu safranin **(E)** and analyzed for connective and mucosal proteases mRNA expression **(F)**. Data are expressed as mean +SD from n>3 experiments; statistical analysis were performed with one-way Anova with Dunnet correction (**p < 0,01).

For the stimulation–restimulation panel (described in **Figure 1A**), BMMCs were differentiated for 6 weeks in complete IL-3 medium and checked for purity by flow cytometry. BMMCs were left untreated or stimulated for 24 hours with 1 μg/ml LPS (LPS from *E. coli* O55:E5, Sigma Aldrich), 10 μg/ml curdlan (*In vivo*gen), IgE/Ag (100 ng/ml DNP) in IL-3–free complete RPMI medium. After the stimulation, supernatants were collected and cells were washed twice with PBS and put back in culture in fresh complete–IL-3 for 6 days. Each cells subset was then re-stimulated with 100 ng/ml LPS, live *C. albicans* yeasts (MOI=1) or IgE/Ag for 24 hours. In some experiments, before the addition of the first stimulus, cells were pre-treated with inhibitors of HDAC: 1nM suberoylanilide hydroxamic acid (SAHA, from Sigma Aldrich) or 10nM Trichostatin (TSA, from Sigma Aldrich).

### Flow Cytometry

0.5 x 10^6^ cells were harvested, washed with PBS and stained for 30 minutes at 4°C in the dark with monoclonal conjugated antibodies. All antibodies were from Biolegend: anti-FcεRI–PE (MAR-1), anti-cKit–PE-Cy7 (ACK2), anti-CD14 PE (Sa14-2), anti-TLR2 PE (6C2), anti-TLR4 APC (SA15-21), anti-Dectin-1 PE (bg1fpj). Cells were then washed twice with PBS and acquired with a FACSCalibur flow cytometer (Becton Dickinson).

### Histochemical Staining

10^4^ BMMCs were cytospin onto SuperFrost glass slides (Menzel) at 300 x g for 3 minutes and allowed to air-dry overnight. For toluidine blue staining, slides were incubated for 5 minutes with a solution of 1% toluidine blue [pH 5] (Sigma Aldrich), rinsed twice in PBS and once in water, then allowed to air-dry and mounted with Micro mount (Diapath). For alcian blue–safranin staining, slides were incubated for 10 minutes with a solution of 0.5% alcian blue (Sigma Aldrich) in 0.3% acetic acid [pH 3], rinsed twice in PBS then incubated for 20 minutes with a solution of 0.1% safranin-O (Sigma Aldrich) in 0.1% acetic acid [pH 4]. Slides were then rinsed and mounted as described above. Images were acquired with a Leica DM750 equipped with a Leica ICC50W camera at a magnification of 63x and processed with Fiji (ImageJ) software.

### Degranulation Assay

BMMCs degranulation response was determined as the percentage of β-hexosaminidase released. 0.5 x 10^6^ BMMCs were incubated in Tyrode’s buffer (10 mM HEPES buffer [pH 7.4], 130 mM NaCl, 5 mM KCl, 1.4 mM CaCl_2_, 1mM MgCl_2_, 5.6 mM glucose, and 0.1% BSA) with or without the addition of 10% FBS, and stimulated with the same number of *C. albicans* yeasts at 37° for the indicated time points. As positive control, 0.5 x 10^6^ BMMCs were sensitized in complete RPMI medium for 3 hours with 1 μg/ml of dinitrophenol (DNP)-specific IgE, then washed twice, resuspended in Tyrode’s buffer and challenged with 100 ng/ml DNP (Sigma-Aldrich). The enzymatic activity of the released β-hexosaminidase was assessed by the cleavage of its synthetic substrate (p-nitrophenyl N-acetyl- glucosamide, Sigma Aldrich) in p-nitrophenol and measuring the p-nitrophenol absorbance at 405nm with a plate spectrophotometer. Results are expressed as the percentage of b-hexosaminidase released over β-hexosaminidase retained in the cytoplasm.

### Cytokine ELISA Assays

Supernatants for cytokine quantitation were collected 24 hours after BMMC stimulation. Supernatants were assessed for TNF-α and IL-6, using specific ELISA kits (eBiosciences) according to the manufacturer’s instructions.

### RNA Extraction and Real-Time PCR Analyses

Cells were lysed with EUROGOLD TriFast (Euroclone) and total RNA extracted according to manufacturer’s instructions. Total RNA was quantified using a NanoDropTM spectrophotometer (ThermoFischer) and retro-transcribed with the SensiFAST™ cDNA Synthesis kit (Bioline). Quantitative qPCR analyses were performed with SYBR Green chemistry (BioRad) using a BioRad CFX96 real-time PCR detection systems. Target genes expression were quantified with the ΔΔCt method using G3PDH (glyceraldehyde 3-phosphate dehydrogenase) as normalizer gene. The un-treated sample with the lowest expression was set to one and used to calculate the fold inductions of all other samples. Primer’s sequence are:

mG3PDH_for TCAACAGCAACTCCCACTCTTCC;
mG3PDH_rev ACCCTGTTGCTGTAGCCGTATTC;
mMCPT-4_for GGGCTGGAGCTGAGGAGATTA;
mMCPT-4_rev GTCAACACAAATTGGCGGGA
mMCPT-2_for AAGCTCACCAAGGCCTCAAC;
mMCPT-2_rev ACACCACCAATAATCTCCTCAG
mMCPT-6_for CGACATTGATCCTGACGAGCCTC;
mMCPT-6_rev ACAGGTGTTTTCCACAATGG
mTNF-alpha_for AGGCACTCCCCCAAAGATG;
mTNF-alpha_rev CCATTTGGGAACTTCTCATCCC
mIL-6_for ACCACTTCACAAGTCGGAGGCTTA;
mIL-6_rev TCTGCAAGTGCATCATCGTTGTTC;
mSOCS3_for GGGAGCCCCTTTGTAGACTT;
mSOCS3_rev CATCCCGGGGAGCTAGT

### Cell Lysis and Western Blot Analyses

BMMCs were stimulated with an equal number of *C. albicans* yeasts or with the indicated stimuli or left untreated for the indicated times. Cells were then incubated for 5 minutes on ice to stop the reaction and centrifuged 300 x g, 5 minutes at 4°C. Before the lysis, BMMCs were washed twice with PBS + phosphatase inhibitors (Active Motif). Cell pellet were lysed in 50 μl NP-40 buffer (25 mM Tris-HCl [pH 7.4], 150 mM NaCl, 1 mM EDTA, 1% NP-40 and 5% glycerol, 1 mM Na3VO4, 50 mM NaF (Sigma Aldrich), and COmplete Mini protease inhibitor cocktail (Roche, usage according to manufacturer)) for 10 minutes on ice. Lysates were then centrifuged at 12000 x g, 4°C for 10 minutes and supernatants were collected and stored at -80 C.

Upon western blot analyses, lysates were diluted with 4x Laemmli buffer and denatured 7 minutes at 95°C. Lysates were then separated on SDS 10% polyacrilamide gels and blotted on a nitrocellulose membrane (Amersham) at 300 V, 250 mA, 4°C for 3 hours. Membranes for phosphoproteins were blocked in TBS-T (20 mM Tris-base [pH 7.6], 150 mM NaCl, 0.05% Tween-20) + 5% BSA while membranes for total proteins were blocked in TBS-T + 5% non-fat dry milk for 1 hour at room temperature. Primary antibodies for phosphoproteins were diluted 1:1000 in TBS-T + 5% BSA and membranes probed overnight at 4°C with gentle agitation. HRP-conjugated secondary antibodies (Pierce) were incubated for 1 hour at room temperature with gentle agitation. Signals were detected using ECL substrates Luminata forte (Millipore) or SuperSignal West Femto (Thermo Scientific) and acquired with a ChemiDoc imaging system (BioRad). Densitometric analyses were performed with the Image Lab 5.2 software (BioRad). Probed membranes were stripped twice for 10 minutes at room temperature in mild stripping buffer (0.2 M glycine, 0.1% SDS (Sigma Aldrich), 1% Tween20, pH 2.2) than blocked in TBS-T + 5% non-fat dry milk for 1 hour at room temperature and reprobed overnight at 4 C with gentle agitation. Antibodies (clones, vendors): actin (C4, BD biosciences), AKT (BD bio- sciences), phospho-AKT S473 (D9E, CST), PI3K p85 (CST), phospho-PI3K p85 p55 Y459 Y199 (CST), p38 MAPK (CST), phospho-p38 MAPK T180 Y182 (CST), PI3K p110d (A-8, Santa Cruz).

### NF-κB Activity

Nuclear protein fractions were obtained as described above and protein concentration was measured with the PierceTM BCA Protein Assay Kit (Thermo Scientific). 20 μg of protein extracts were used for the quantification of NF-κB p65 and p50 activity using the TransAM™ NF-kB kit (Active Motif) according to manufacturer’s instructions.

### DNA Methylation Analysis

CpG methylation was determined by either pyrosequencing or real-time methylation specific PCR (rt-MSP). For each condition, gDNA was extracted and bisulphite-converted from up to 10^5^ cells using the EpiTect Fast Bisulfite Conversion kit (Qiagen). ssDNA was quantified with NanoDrop 2000 and 20 ng were used to produce PCR amplicons. TNF-α promoter methylation was determined by pyrosequencing: PCR products from bisulphite-converted DNA were sequenced using PyroMark Q24 ID (Qiagen) while the data was analyzed with PyroMark software. In order to perform the analysis, the selected region (mm10 chr17:35201591-35202581) was divided into two subregions (primer listed in **Supplemental Material S2**). Both regions were amplified with 45 cycles: region 1 Ta=50°C while region 2 Ta= 55°C.

SOCS3 promoter methylation was determined instead by rt-MSP. The methylation status was quantified calculating the difference between unmethylated and methylated Ct values (uCt – mCt): the smaller the ΔCt value is, the greater will be the proportion of methylation in the region (14). All primers used for methylation analysis are listed in in **Supplemental Material S2**.

### Statistical Analyses

Unless otherwise indicated, results are expressed as mean ± standard deviation (SD) of at least three experimental replicates. Data were analyzed using paired Student t-test or ordinary one-way ANOVA tests with Dunnet correction (GraphPad Prism v6). *p < 0.05, **p < 0.01, ***p < 0.001.

## Results

### Different Priming Affect MCs Phenotype

To assess whether long-lasting trained immunity or tolerance could be induced in BMMCs, cells were differentiated for 5 weeks in IL- 3 media and then primed for 24 hours by stimulation with either LPS, curdlan (a dectin-1 agonist consisting of β-(1,3)-linked glucose residues), IgE/Ag or left untreated (RPMI). BMMCs were then washed and put back in culture in fresh IL-3 media to allow their recovery. After 6 days, each cell subset was investigated for proliferation rate and phenotype, then subjected to a second stimulation with the same or a different stimulus (LPS, IgE/Ag or live *Candida albicans*) and investigated for their responsiveness (**Figure 1A**).

As shown in **Figure 1B**, during the 6 days of resting time all the cells kept proliferating at a low rate, and the cell viability (determined by trypan-blue exclusion) was found to be comparable for all the stimulations (**Figure 1B**). The size of the cells and their granularity did not change after the stimulation, as indicated by the almost identical levels of forward scatter (FSC) and side scatter (SSC). Similarly, FcεRI and cKit levels remained constant after the resting time (**Figure 1C**) indicating that BMMCs did not lose their cellular identity after the priming. Moreover, no differences in Dectin-1, CD14, TLR2 and TLR4 protein expression were detected between unstimulated and differently primed BMMCs (**Figure 1C**).

To define whether MCs priming was responsible for a phenotypic change in MCs, 6 days after the pre-stimulation BMMCs were stained with toluidine blue and alcian blue–safranin to visualize granules. BMMCs granule content and metachromasia were comparable between the four conditions (**Figures 1D, E**). Interestingly, BMMCs that degranulated after the first challenge with IgE/Ag, recovered most of their granules and stained metachromatically with toluidine blue (**Figure 1D**).

To assess whether the expression profile of MC proteases was influenced by the priming with different stimuli, we measured the gene expression levels of connective and mucosal MC proteases in BMMCs 6 days after the pre-stimulation with LPS, curdlan, IgE/Ag and RPMI. Curdlan and IgE/Ag pre-stimulation did not modify the mRNA expression of MC proteases, while LPS priming remarkably increased *MCPT-4* (but not *MCPT-2* and *MCPT-6*) expression, -compared to untreated cells (**Figure 1F**).

These results suggest that the stimulation of BMMCs with different ligands do not alter the expression levels of TLR2, TLR4, CD14, and Dectin-1. However, BMMCs stimulated with curdlan or IgE/Ag conserve their mucosal-like phenotype, while MC priming with LPS stably modifies the composition of granule proteases with an increase in *MPCT-4* gene expression.

### MCs Priming Differently Affects the Degranulation and Cytokine Response to Secondary Inflammatory Stimuli

**Figure 2:**
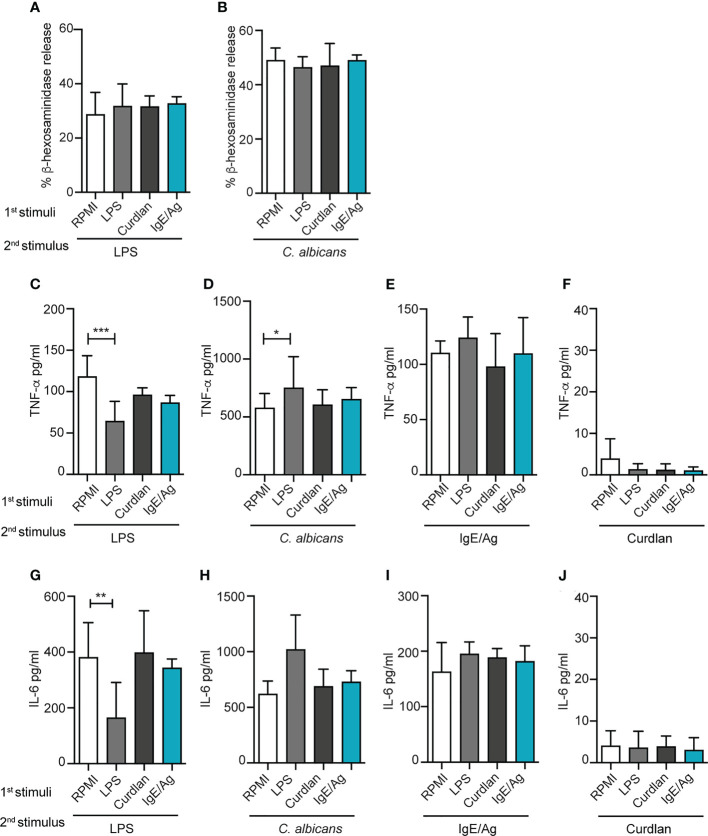
Effect of different priming on MC degranulation and cytokine release response to a secondary challenge. Degranulation of BMMCs primed with LPS (1mg/ml), curdlan (100mg/ml), IgE/Ag or RPMI was determined 6 days after cell recovery as percentages of released β-hexosaminidase in response to 30 min of stimulation with IgE/Ag **(A)** or ionomycin **(B)**. BMMC primed with RPMI, LPS (1mg/ml), curdlan (100mg/ml) or IgE/Ag were subjected to a second stimulation with LPS **(C–G)**, live *C. Albicans* **(D–H)**, IgE/Ag **(F–I)** or curdlan **(E–J)**. TNF-α **(C–F)** and IL-6 **(G–J)** levels were detected in culture supernatants after 24 hours. Data are expressed as mean +SD from n>4 experiments; statistical analysis were performed with one-way Anova with Dunnet correction (*p < 0,05 **p < 0,01; ***p < 0,001).

To address whether the pre-stimulation of MCs with different stimuli could influence their ability to mount an effective degranulation response, we quantified the amount of β-hexosaminidase released by pre-stimulated MCs in response to anaphylactic stimuli. We first studied the degranulation response to the classical Ag challenge of IgE sensitized MC: BMMC were pre-stimulated with LPS, curdlan or IgE/Ag, allowed to rest for 6 days, then sensitized with IgE and finally challenged with the specific antigen for 30 minutes. As shown in **Figure 2A**, the pre-stimulation did not affect the amount of β-hexosaminidase released following the aggregation of the high-affinity receptor for IgE. Moreover, similar degranulation responses were observed by pre-stimulated BMMCs once challenged for the same short time with the Ca^++^ ionophore ionomycin (**Figure 2B**). These results suggest that the early events that characterize the anaphylactic reaction in BMMCs are not influenced by previous conditioning of the cells.

The effect of the pre-stimulation on cytokine production induced by a second stimulation was then investigated by ELISA (**Figures 2C–J**). BMMC primed for 24h with RPMI, IgE/Ag, LPS or curdlan, were re-stimulated, after 6 days, with IgE/Ag, a 10-fold lower dose of LPS or live *C. albicans* yeasts for 24 hours (as depicted in **Figure 1A**), and the levels of TNF-α and IL-6 secretion were measured.

Single stimulations with IgE/Ag, LPS or live *C. albicans* yeasts induced the secretion of comparable levels of cytokines, while curdlan caused only a minimal release of both TNF-α and IL-6 (**Supplemental Figure S1**). Interestingly, when we investigated the cytokine secretion induced in differently primed BMMC by a second stimulation, we observed diverse responses depending both on the first and the second stimulus. As expected, the priming with LPS impaired BMMCs response to a secondary challenge with LPS as demonstrated by the lower release of TNF-α (**Figure 2C**) and IL- 6 (**Figure 2G**). On the other side, priming with curdlan or IgE/Ag did not affect MCs’ responsiveness to secondary LPS challenge (**Figures 2C, G**).

To determine whether LPS priming could induce cross-tolerance also against non-TLR4 stimulations, BMMCs were restimulated either with live *C. albicans* yeasts or IgE/Ag. Strikingly, priming with LPS induced a stronger response to *C. albicans* compared to RPMI control, enhancing the release of TNF-α (**Figure 2D**) but not of IL-6 (**Figure 2H**). As for LPS, the response against *C. albicans* challenge by MCs primed with curdlan or IgE/Ag was comparable with the control (**Figures 2D, H**). By contrast, the pre-stimulation with curdlan did not affect the responsiveness to secondary stimuli. Notably, the first stimulation with LPS, curdlan and IgE/Ag did not influence the response to stimulation with the classical anaphylactoid stimulation IgE/Ag that was comparable between all the four groups, in terms of release of both TNF-α and IL-6 (**Figures 2E, I**). In our system, curdlan stimulation didn’t exert any relevant training or tolerance effect (**Figures 2F, J**).

Taken together, our results indicate that LPS pre-stimulation can induce an opposite, dual response in MCs depending on the secondary trigger received by the cells.

### LPS Priming Modulates Cytokines and Proteases Expression at mRNA Level

**Figure 3:**
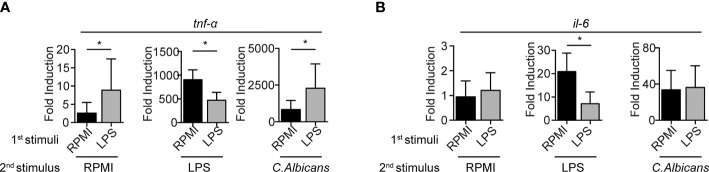
mRNA cytokine expression levels are impaired in LPS-primed BMMCs. mRNA expression of *TNF- α* **(A)** and *IL-6* **(B)** were measured by rt-PCR on LPS-primed BMMC after 3 hours of stimulation with LPS (1mg/ml), live *C. Albicans* (MOI=1) or RPMI. Data are expressed as mean +SD from n=3 experiments. Statistical analysis were performed with paired Student t-test (*p < 0,05).

To assess whether BMMCs conditioning with LPS correlates with cytokines mRNA transcription, qPCR analyses were performed 3 hours post-restimulation. Consistent with the protein release detected by ELISA, the stimulation with a second dose of LPS resulted in the impaired expression of the transcripts for both TNF-α and IL-6 in LPS-primed BMMCs (**Figure 3**). Moreover, stimulation of LPS-primed BMMCs with *C. albicans* yeast caused an increased expression of *TNF-α* (**Figure 3A**) but not of *IL-6* (**Figure 3B**). Interestingly, LPS-primed BMMCs showed an increased basal transcription of *TNF-α*, 3.3 times higher than controls. Stimulation with *C. albicans* induced a 300-fold increase in *TNF-α* transcription levels in both LPS-primed BMMCs and controls, which were found to be 2.7 times higher in LPS-primed cells (**Figure 3A**). Taken together, these results suggest that LPS-priming induce a higher basal transcription for *TNF-α* but not *IL-6*, and that *C. albicans* challenge likely has the same effect on both LPS-primed and control cells.

### LPS Conditioning Impairs the PI3K-AKT Pathway

**Figure 4:**
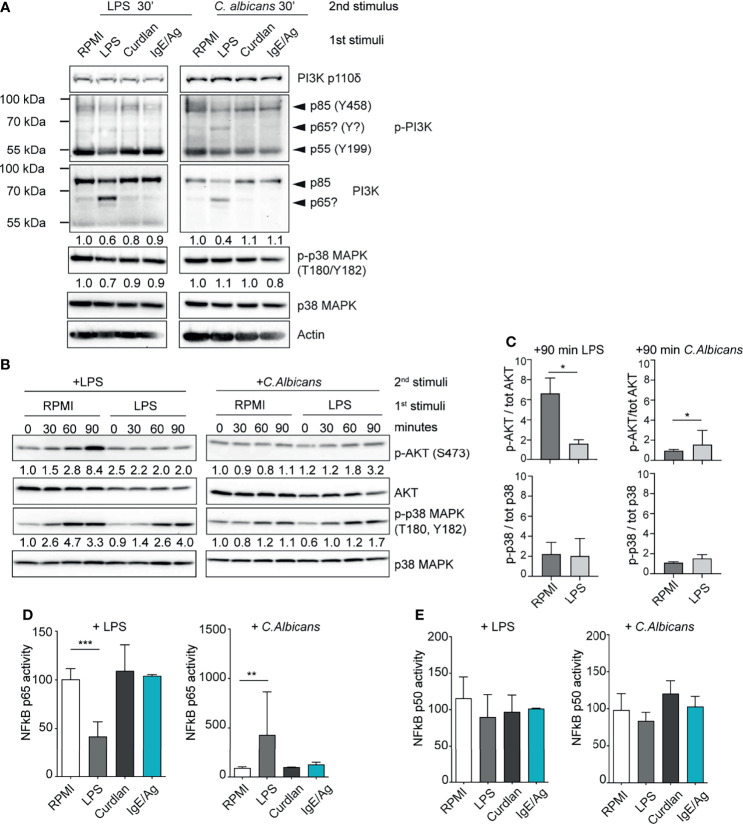
LPS-primed MCs differently activate the PI3K-AKT pathway. **(A)** Western Blot (WB) analysis of PI3K subunits and of phosphorylated p38 in differently primed-BMMC restimulated for 30 minutes with LPS or live *C. Albicans* (MOI=1). Numbers indicate the results of densitometric analyses calculated over endogenous protein expression. **(B)** Time-course WB analysis of phosphor Akt and phospho p38 in LPS primed-BMMC restimulated with LPS or live *C. Albicans* (MOI=1). Numbers indicate the results of densitometric analyses calculated over endogenous protein expression normalized versus t=0. **(C)** Mean + SD of densitometric analysis of n=3 independent experiments are reported. Statistical analyses were performed with paired Student t-test (*=p<0,05). NF-kB p65 **(D)** and p50 **(E)** activity in LPS primed BMMC was determined after 30 minutes form addition of the second stimuli. Results were expressed as fold percentages over control (RPMI). Mean + SD of n=3 independent experiments are reported. Statistical analysis were performed with one-way Anova with Dunnet correction (*p < 0,05 **p < 0,01; ***p < 0,001).

In MCs, the regulation of TNF-α and IL-6 production during TLR-mediated stimulation involves the activation of the PI3K pathway (15). This pathway is of great importance also for MCs development and for the activation of the complimentary FYN-GAB2-PI3K signaling cascade during IgE/Ag activation. Seen that PI3K seems to play a dual role depending on the cellular context (16), the modulation of PI3K, AKT and p38 MAPK pathways in primed BMMCs was evaluated (**Figure 4**).

Strikingly, LPS-primed BMMCs showed a decreased level of total PI3K p85 and lower levels of PI3K p85 and p55 phosphorylation following *C. albicans* infection (**Figure 4A**). This phenomenon correlated with the expression of a putative isoform (or truncated version) of PI3K with an approximative molecular weight of 65 kDa (**Figure 4A**). This putative isoform was recognized both by the anti-p85 full-length antibody and to a lesser extent by the anti-phospho-PI3K antibody which recognizes the phosphorylated Tyr458 of p85 and Tyr199 of p55 (**Figure 4**). LPS-primed MCs displayed also a reduced phosphorylation of p38 MAPK 30 minutes after the re-stimulation with low-dose LPS (**Figure 4A**). Conversely, the same cells showed no impairment of p38 phosphorylation after 30 minutes of *C. albicans* challenge. All the cell expressed comparable levels of the catalytic subunit PI3K p110d (**Figure 4A**).

This phenomenon prompted us to compare the kinetics of AKT activation level during LPS- and *C. albicans*-induced activation of primed MCs (**Figures 4B, C**). Control BMMCs (RPMI) showed a strong activation of AKT already after 60 minutes of stimulation with second dose of LPS, while it was completely abolished in LPS-primed MCs. AKT activation in control cells correlated with an earlier and more sustained activation of p38 MAPK (**Figures 4B, C**). On the other side, *C. albicans* failed to induce the activation of AKT in both the cell populations but LPS- primed MCs displayed an enhanced activation of p38 MAPK.

To further characterize the nature of the ambivalent behavior of LPS- primed MCs, we looked at the activation status of the NF-kB transcription factor. During LPS restimulation NF-kB p65 activity was reduced in LPS-primed MCs while after challenge with *C. albicans* LPS-primed cells displayed an increase of NF-kB p65 activity (**Figure 4D**). Interestingly, NF-kB p50 activity was found to be unaltered by cell priming (**Figure 4E**).

These results indicate that the differential response following the restimulation of LPS-primed MCs with LPS or *C. albicans* is likely due to an altered activation of the downstream PI3K-AKT-p38 signaling pathway, which resulted in the impaired activation of NF-κB.

### LPS Tolerance Is Partially Dependent on Histone Acetylation

Several studies demonstrated that the induction of trained immunity in myeloid cells is dependent on epigenetic mechanism (17), including modification in the DNA methylation profile and in chromatin accessibility.

**Figure 5:**
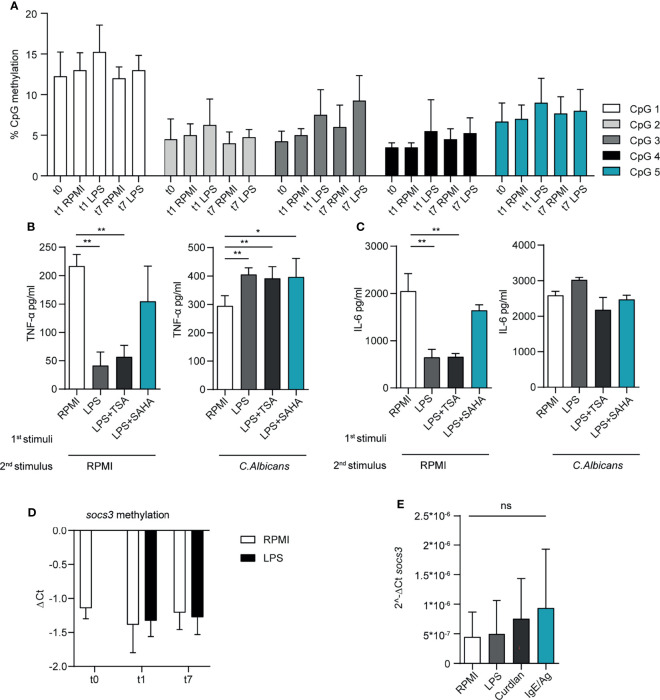
LPS priming affects the epigenetic landscape in MCs. **(A)** CpG methylation at TNF-α promoter region. A total of five CpG sites were analysed in the selected region, their level of methylation was determined by pyrosequencing. Mean + SD of n=3-5 independent experiments are reported. BMMC were primed with LPS (1μg/ml) in presence of HDAC inhibitors (SAHA 1nM; TSA 10nM), then washed, re-stimulated and analysed for TNF-α **(B)** and IL-6 **(C)** secretion upon second stimulation with LPS or *C. albicans*. Mean + SD of n=3 independent experiments are reported. **(D)** SOCS3 promoter CpG methylation was determined by real-time MSP. Methylation was calculated as difference among Ct values of unmethylated and methylated region. Mean + SD of n=2-6 independent experiments are reported. **(E)** *SOCS3* mRNA expression in BMMC at day 7, i.e. 6 days after the first stimulation with LPS, curdlan and IgE/Ag. Mean + SD of n=3 independent experiments are reported. Statistical analysis were performed with one-way Anova with Dunnet correction (*p < 0,05 **p < 0,01; ***p < 0,001 ns, not significant).

To demonstrate if similar mechanisms also happens in MCs, at first, we investigated the CpG methylation profile of the TNF-α promoter in BMMCs one week after pre-stimulation with RPMI or LPS by pyrosequencing. A total of five CpG sites were analyzed in the selected region, however, we did not detect significant differences in their level of methylation. A similar level of methylation was observed in all the CpG after the first stimulation with LPS and curdlan, as well as after the recovery period before the second stimulation (**Figure 5A**).

We then addressed the possibility that chromatin accessibility in primed MCs was differently influenced by pre-stimulation with by LPS. Therefore, we pretreated BMMC with histone deacetylase (HDAC) inhibitors, namely suberoylanilide hydroxamic (SAHA) and Trichostatin (TSA), for 1 hour before the addition of LPS or RPMI. After 6 days, we investigated the ability of these BMMCs to release cytokines in response to a re-stimulation with LPS or live *C. albicans*. We observed that both the inhibitors partially restored the secretion of TNF-α (**Figure 5B**) and IL-6 (**Figure 5C**) after LPS stimulation when compared with untreated BMMC, but failed to affect the cytokine secretion in response to *C. albicans* restimulation. These results indicate that HDAC inhibitors can interfere with cytokine production after MC activation through TLR4.

In immune cells, including MCs, the expression of cytokines is negatively controlled by the activity of the cytokine-suppressor-proteins (SOCS) (18–20). To understand whether SOCS protein could be involved in the different responses of primed MC to re-stimulation, we investigated the level of mRNA expression of SOCS3 and the methylation profile of SOCS3 promoter by rt-MSP, in BMMCs pre-stimulated with LPS. rt-MSP showed that the methylation profiles of SOCS3 promoter was comparable between unstimulated cells (RPMI) and MCs primed with LPS or curdlan, either after the first stimulation and the recovery period (**Figure 5D**). Moreover, quantitative PCR performed on MCs at day 7 (i.e. 6 days after the first stimulation and before the addition of the second stimulus) showed similar levels of SOCS3 expression in all primed cells, excluding that the observed reduction in cytokine expression was due to a different activity of SOCS3 (**Figure 5E**).

Taken together, these results suggest that histone acetylation by HDAC, but not chromatin methylation, might be partially responsible for the reduced release of TNF-α and IL-6 following LPS restimulation in LPS-primed BMMCs.

### LPS Tolerance Is Long-Lasting and Induced Early in MCs Differentiation

**Figure 6:**
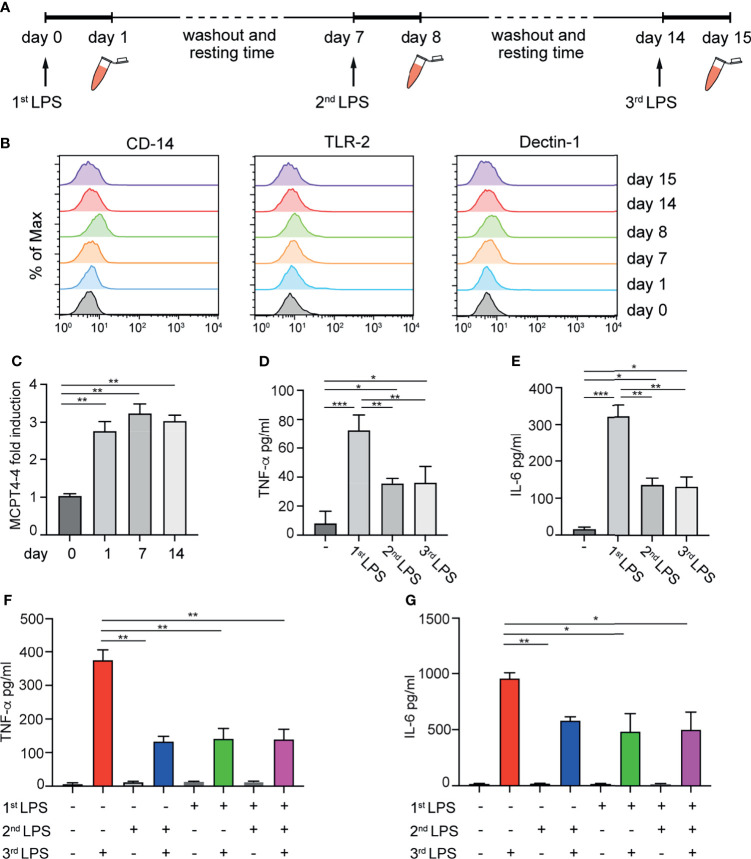
Effect of repetitive LPS stimulation on BMMCs. Schematic representation of experimental protocol: 4-weeks old BMMCs were treated for 24h with LPS once a week for three weeks **(A)**. Surface marker expression **(B)** and *MCPT-4* mRNA expression **(C)** were evaluated at indicated times. The levels of TNF-α **(D)** and IL-6 **(E)** released by BMMC after the first, the second and the third dose of LPS are shown. The levels of TNF-α **(F)** and IL-6 **(G)** released by BMMC treated with one or more doses of LPS have been compared. Data are means +SD. Statistical analysis were performed with one-way Anova with Dunnet correction (*p < 0,05 **p < 0,01; ***p < 0,001).

Considering that MCs *in vivo* displays extreme heterogeneity and variability depending on the local microenvironment, the possibility to induce the maturation and skewing of immature MC towards a tolerant or pro-inflammatory phenotype could be relevant. Therefore, having established that mature BMMCs can develop a long-lasting immunological memory, we decided to investigate the effect of repetitive stimulation of MCs with LPS at early stages of maturation. 4-weeks old BMMCs were challenged for 24 h with LPS, washed and put in culture for 6 days in IL-3 containing medium. The same treatment was repeated once a week for three weeks as depicted in **Figure 6A**. BMMCs were investigated for their phenotype, the expression of *MCPT-4*, and the release of TNF-α and IL-6. As shown in **Figure 6B**, multiple stimulations with LPS did not modify the expression of surface markers, neither of TLR-2, CD14 and Dectin-1 (**Figure 6B**). However, the first stimulation with LPS induced an increased expression of *MCPT-4* mRNA after the first treatment that persisted up to 14 days (**Figure 6C**). Analysis of the cytokine released in response to repeated LPS stimulation demonstrated that the priming of BMMC with the first dose of LPS induced a tolerant phenotype that lasted for more than 2 weeks. Indeed, as shown in **Figures 6D, E**, LPS-primed BMMCs challenged with a second and a third dose of LPS did not recover the ability to fully release cytokines and their hypo-responsiveness was maintained. This effect was also confirmed by comparing the levels of cytokines released by BMMCs treated with one or more doses of LPS. LPS-primed BMMCs allowed to recover and exposed to the second dose of LPS after 2 weeks (green bar) still maintained their hyporesponsiveness and released lower amounts of TNF-α (**Figure 6F**) and IL6 (**Figure 6G**) compared to MCs challenged with a single LPS dose (red bar).

These results demonstrate that LPS tolerance in LPS-primed MCs can last for at least two weeks and can be induced already in the early stages on MC differentiation.

## Discussion

The fact that MCs are tissue-resident cells which can survive for up to 12 weeks, and that their terminal differentiation is highly influenced by tissue-specific stimuli, renders these cells possibly implicated in innate immune memory.

In the last decades, some studies on the nature of endotoxin tolerance demonstrated memory-like traits also in MCs. Initially, it has been reported that LPS stimulation induced endotoxin tolerance in BMMCs through the upregulation of the phosphatase SHIP (but not of SHIP2 nor PTEN) by the autocrine effect of LPS-induced release of TGF-β (11). More recent studies demonstrated the ability of MCs to respond to LPS by releasing pro-inflammatory TNF-α and IL-6 in a p38-dependent manner, and that the release of these cytokines was severely decreased upon a secondary stimulation with LPS or other TLR2 agonists, suggesting that endotoxin tolerance could also induce cross-tolerance against different stimuli (18, 21). A recent report by Poplutz et al. demonstrated that after a second stimulation with LPS, MCs displayed a decreased nuclear translocation and DNA binding ability of the canonical NF-kB p65/p50 heterodimer (17). The latter phenomenon was due to the constitutive presence of the suppressive marker H3K9me3 on the promoters of TNF-α and IL-6 which inhibited NF-kB binding to those promoters. These repression markers were transiently lost during the initial LPS stimulation (thus allowing NF-kB binding and gene transcription) but remained unchanged during the second LPS challenge. According to previous reports, short pre-treatment with LPS did not affect TNF-α and IL-6 release during IgE/Ag cross-linking, possibly due to the involvement of Ca^++^-dependent NFAT activation (13, 22). Nevertheless, all these studies were restricted to LPS pre-stimulation and were limited to a rapid *in vitro* restimulation after cell priming.

To address more in detail the possibility that different types of environmental signals might influence MC reactivity to subsequent and repetitive stimulations, we set up an experimental model which allowed the cells to rest for 6 days between the first stimulation and the subsequent restimulation. We then systematically compared the ability of different signals to either train or induce tolerance on MCs.

In line with previous findings (13, 22), the response to the classic anaphylactoid IgE/Ag stimulation was not affected by the priming with LPS, both in terms of TNF-α, IL-6 and β-hexosaminidase release. Similarly, IgE/Ag priming of BMMCs did not modify the response to a second stimulation with LPS, *C. albicans* or to the re-engagement of the FcεRI by IgE and antigen. Unlike with monocytes and macrophages, priming of MCs with the Dectin-1 ligand curdlan did not boost cytokine secretion in response to LPS or *C. albicans* stimulation and, therefore, failed to induce a training effect in MCs. Other authors demonstrated the ability of curdlan to induce cytokine productions in MCs. However, in our hands curdlan induced only a minimal release of IL-6 and TNF-α in BMMC and seems to be unable to conditionate cells to further stimulation. This could be due to different handling procedures between laboratories, nevertheless, besides curdlan, we tried different ligands of Dectin, such as β-glucan or WPG (data not shown), but none of these has been found to induce a strong BMMC response or to influence the MC reactivity against further stimulation.

As expected, the impaired release of TNF-α and IL-6 showed by LPS-primed cells in response to LPS confirmed that MCs became tolerant to a second dose of LPS, and suggests that endotoxin tolerance can be maintained for at least 6 days. Surprisingly, the pre-stimulation of BMMCs with LPS did not modify their ability to release cytokines in response to a challenge with *C. albicans*. As a matter of fact, restimulation of LPS-primed cells with *C. albicans* caused a higher release of TNF-α compared to naïve BMMCs, suggesting that LPS priming can induce both tolerance and training depending on the secondary stimuli. We excluded that the altered responses of re-stimulated BMMC could be mediated by the differential expression of pattern recognition receptors involved in the recognition of *C. albicans* or LPS, as the expression levels of TLR4, TLR2, and Dectin-1 were comparable between primed and naïve cells.

Interestingly, BMMCs primed with IgE/Ag or curdlan maintained the same mucosal-like phenotype of naïve unprimed BMMCs, while the exposure to a single dose of LPS induced an increased mRNA expression of the connective-tissue protease MCPT-4 that persisted for 6 days. MCPT-4 is a MC protease normally found in the cytoplasm of connective-tissue MCs, but it is also well-known for the anti-inflammatory role that can exert. For example, this protease was found to be involved in the degradation of MC-derived TNF-α in a murine model of sepsis, as MCPT-4-deficient mice exhibited increased levels of intraperitoneal TNF-α and higher numbers of peritoneal neutrophils (23). Moreover, unlike mucosal MCs, connective-tissue MCs are known to express high levels of TGF-β during fungal infections, thus contributing to mucosal immune tolerance (24). In addition, MCPT-4 is required for the cleavage and activation of TGF-β which, in turn, can activate a Th17 response that is known to be involved in fungal immunity (25, 26). Therefore, MCPT-4 could contribute on one hand to the tolerogenic response of LPS pre-stimulated MC that show a reduced TNF-α release after a second LPS stimulation, and on the other hand, it could be involved also in the enhanced response to *C. Albicans* infections. Notably, the upregulation in MCPT-4 mRNA expression and the reduced secretion of cytokine in response to the second dose of LPS was also found in BMMCs two weeks after the first stimulation with LPS, and in BMMCs exposed to repetitive LPS expositions, suggesting that a single, early priming of MCs can affect their behavior for a long time.

Although it is known that LPS can induce endotoxin tolerance or training depending on the dose used for cell priming (27), here we report an ambivalent role for LPS in the induction of both tolerance and training depending on the secondary challenge. This phenomenon is made possible by the result of the combined rewiring of intracellular signaling cascades and epigenetic changes in the regulating accessibility to the loci of TNF-α. On one hand, LPS-priming induced long-term tolerance to endotoxin by impairing the PI3K-AKT pathway possibly through the modulation of PI3K p85 expression. The lack of AKT activation correlated with an impaired p38 phosphorylation and NF-kB p65 activity, finally leading to a reduced expression of TNF-α and IL-6. On the other hand, LPS-priming induced a higher basal transcription of the *Tnf-α* gene (**Figure 3A**). We believe that this phenomenon could be the result of improved chromatin accessibility on TNF-α regulating regions or due to the constitutive presence of active enhancers/co-activators. Inhibition of HDAC activity partially restored the ability of LPS primed cells to activate a transcriptional response to second stimulation with LPS. Interestingly, inhibition of HDAC activity did not modify the amount of cytokine produced by MC in response to *C. albicans* yeast independently on the first stimulus. These results suggest that altered HDAC activity in LPS-primed cells partially accounts for the repressed transcription of inflammatory genes and that stimulation with live *C. albicans* is likely to be sufficiently different to activate transcription in tolerant cells. These results do not rule out the possibility that the combination of specific patterning of histone modifications, including methylation, phosphorylation, and acetylation, provide integral information for enhancing transcription. At the same time, it must be considered that these cells developed a profound rearrangement of the intracellular signaling network involved in TLR4 responses which could override the higher basal TNF-α transcription. Further proof of this hypothesis is given by the pattern of IL-6 expression: its reduction during LPS-restimulation indicates that the impaired signaling pathway controlling TNF-α expression also affects other pro-inflammatory cytokines. On the other side, restimulation with *C. albicans* did not affect IL-6 expression in primed cells, suggesting that the control of *TNF-α* transcription might be governed by gene-specific chromatin modification rather than by the rewiring of the signaling cascades activated by the fungus.

Taken together, our results suggest that LPS challenge induce long-term memory in MCs promoting, at the same time, tolerance towards further LPS restimulation and immune training during *C. albicans* infection. However, the exact mechanisms orchestrating a tolerogenic rather than trained response in primed MCs remains unclear, as it appears to rely on a multi-layered process that most likely involves receptor signals rewiring, chromatin modification, and gene reprogramming. Additional efforts will be required to elucidate the nature of this ambivalent phenomenon mediated by LPS. By expanding the signaling pathway involved in LPS and fungal sensing, and characterizing the putative epigenetic mechanisms controlling TNF-α expression it will be possible to describe the roles of MCs priming in both homeostatic and pathological conditions.

## Data Availability Statement

The original contributions presented in the study are included in the article/**Supplementary Material**. Further inquiries can be directed to the corresponding author.

## Ethics Statement

The animal study was reviewed and approved by OPBA of University of Udine.

## Author Contributions

MZ, CS, CP, and BF conceived and designed the experiments. MZ, BF, CS, and ST performed the experiments. AA cultured the fungus and performed some experiments. MZ, CS, and ST analysed the data. MZ, CS, and BF wrote the manuscript. CP and BF supervised the study. All authors read and approved the final manuscript.

## Funding

This work was supported by Associazione Italiana Ricerca sul Cancro (AIRC) under grant IG 2014 N.15561 and Progetti di Ricerca di Interesse Nazionale (PRIN) under grant 2015YYKPNN_003 to CP. MZ was supported by the European Regional Development Fund – Project Support of MSCA IF fellowships at FNUSA-ICRC (No CZ.02.2.69/0.0/0.0/19_074/0016274).

## Conflict of Interest

The authors declare that the research was conducted in the absence of any commercial or financial relationships that could be construed as a potential conflict of interest.

## Publisher’s Note

All claims expressed in this article are solely those of the authors and do not necessarily represent those of their affiliated organizations, or those of the publisher, the editors and the reviewers. Any product that may be evaluated in this article, or claim that may be made by its manufacturer, is not guaranteed or endorsed by the publisher.
