# Supplementary material for: LPS Guides Distinct Patterns of Training and Tolerance in Mast Cells
Source: Front Immunol. 2022 Feb 17;13:835348. doi: 10.3389/fimmu.2022.835348 (PMC8891506; doi:10.3389/fimmu.2022.835348)

Supplemental Material

S1. Level of cytokines released after 24 h by a single stimulation
TNF-a (A) and IL-6 (B) levels were detected in culture supernatants of BMMC stimulated for 24 hours with LPS (1mg/ml), curdlan (100mg/ml), IgE/Ag or left un-treated (RPMI). N=3; statistical analysis were performed with one-way Anova with Dunnet correction (*=p<0,05 **=p<0,01; ***=p<0,001).

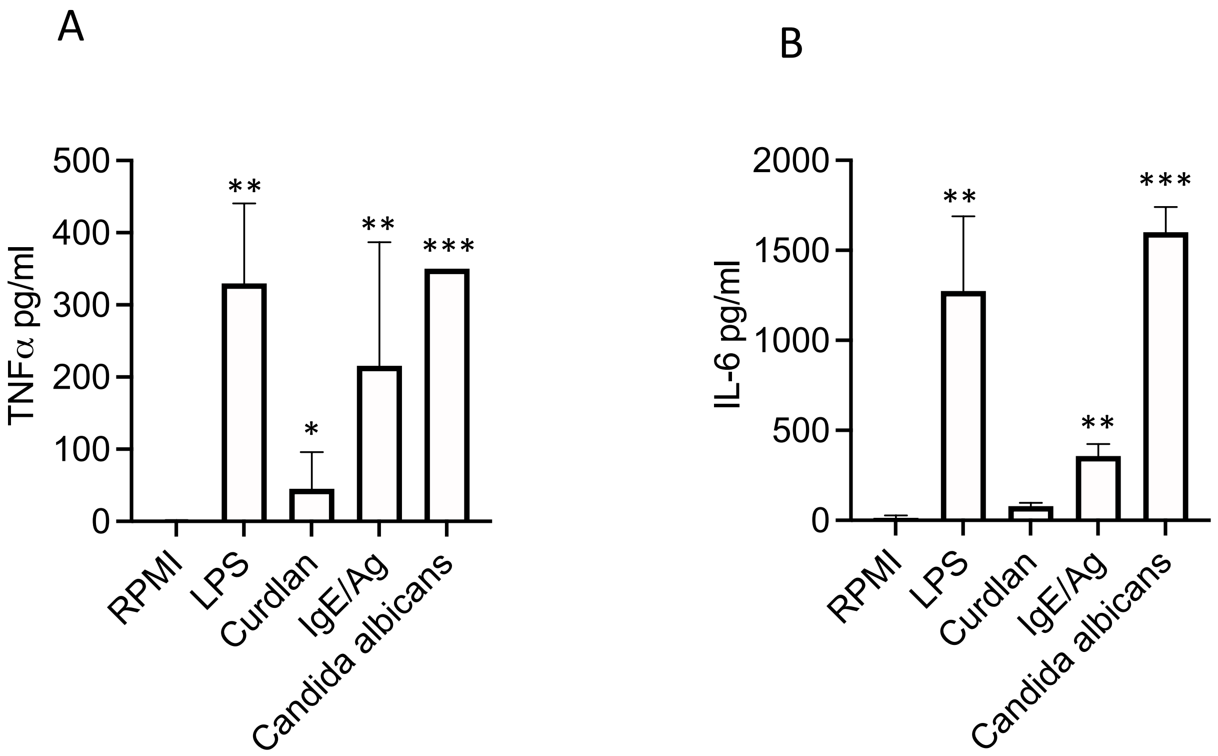

Supplement: Supplementary file 1 [file DataSheet_1.pdf]
