# Supplementary material for: LPS Guides Distinct Patterns of Training and Tolerance in Mast Cells
Source: Front Immunol. 2022 Feb 17;13:835348. doi: 10.3389/fimmu.2022.835348 (PMC8891506; doi:10.3389/fimmu.2022.835348)
Supplement: Supplementary file 2 [file DataSheet_2.pdf]

# Supplemental Material

## S2. List of the primers used for methylation analysys

| Region | Amplification Primer | Sequencing primer |
|---|---|---|
| Region 1 TNF-α | For: TTTTTTATATGAGATTATGG (biotinylated)<br><br>Rev: ACTTCTACTAACTAACTATAC | S1:CCTTTATAACCCTTAAAAAAAAA<br><br>S2: ACTTCTACTAACTAACTATACAA |
| Region 2 TNF-α | For: GAGGGAGAATAGAAATTTTAGAA<br><br>Rev: TAAAATTCTAAAAACCCCCCAT (biotinylated) | S1: AGGTTTTGTTTTTTTTATTTATTG<br><br>S2: ATTATGAGTATAGAAAGTATGAT |

| Target | Primer name | Primer sequence | Application | Note |
|---|---|---|---|---|
| TNF-α chr17:35201591-35202581 | mTNFpromREG1_For (biotinylated) | TTTTTTATATGAGATTATGG | Region1 PCR amplification for pyrosequencing | The region analysed was divided in two fragments (Region 1 and 2) and each fragment was sequenced in two different round (sequence 1 and 2) |
| | mTNFpromREG1_Rev | ACTTCTACTAACTAACTATAC | | |
| | Region1 Sequence1 | CCTTTATAACCCTTAAAAAAAAA | Sequencing primer for Region 1 | |
| | region1 Sequence2 | ACTTCTACTAACTAACTATACAA | | |
| | mTNFpromREG2_For | GAGGGAGAATAGAAATTTTAGAA | Region2 PCR amplification for pyrosequencing | |
| | mTNFpromREG2_Rev (biotinylated) | TAAAATTCTAAAAACCCCCCAT | | |
| | Region2 Sequence1 | AGGTTTTGTTTTTTTTATTTATTG | Sequencing primer for Region 1 | |
| | Region2 Sequence2 | ATTATGAGTATAGAAAGTATGAT | | |
| SOCS3 chr11:117967652-117968576 | meth_SOCS3_for1 | GTTGGGTTTGAGATACGGTT | Primer for methylated fragments | |
| | meth_SOCS3_rev1 | CGCGACGATAACTACAACTACTTC | | |
| | unMeth_SOCS3_for1 | GGTGGTTGGGTTTGAGATATG | Primer for unmethylated fragments | |
| | unMeth_SOCS3_rev1 | AATCTACACAACAATAACTACAACTACTTC | | |
